# Effect of maternal age and body mass index on induction of labor with oral misoprostol for premature rupture of membrane at term: A retrospective cross-sectional study

**DOI:** 10.1515/med-2023-0747

**Published:** 2023-07-04

**Authors:** Gianfranco Sfregola, Pamela Sfregola, Federico Ruta, Federica Zendoli, Alessandra Musicco, Simone Garzon, Stefano Uccella, Andrea Etrusco, Vito Chiantera, Sanja Terzic, Andrea Giannini, Antonio Simone Laganà

**Affiliations:** Department of Obstetrics and Gynecology, “Dimiccoli” Hospital, 76121 Barletta, Italy; Health Agency BAT, General Direction, 76123 Andria, Italy; Department of Obstetrics and Gynecology, Hospital of Bisceglie, 76011 Bisceglie, Italy; School of Nursing Sciences, University of Foggia, 71122 Foggia, Italy; Department of Obstetrics and Gynecology, AOUI Verona, University of Verona, 37129 Verona, Italy; Unit of Gynecologic Oncology, ARNAS “Civico – Di Cristina – Benfratelli”, Department of Health Promotion, Mother and Child Care, Internal Medicine and Medical Specialties (PROMISE), University of Palermo, 90133 Palermo, Italy; Department of Medicine, School of Medicine, Nazarbayev University, 010000 Astana, Kazakhstan; Department of Gynecological, Obstetrical and Urological Sciences, Sapienza University of Rome, 00185 Rome, Italy

**Keywords:** premature rupture of membrane, age, body mass index, labor induction, cervical ripening, oral misoprostol

## Abstract

The aim of this study was to evaluate the effect of maternal age and body mass index (BMI) on induction of labor with oral misoprostol for premature rupture of membrane (PROM) at term. We have conducted retrospective cross-sectional study, including only term (37 weeks or more of gestation) PROM in healthy nulliparous women with a negative vaginal-rectal swab for group B streptococcus, a single cephalic fetus with normal birthweight, and uneventful pregnancy that were induced after 24 h from PROM. Ninety-one patients were included. According to the multivariate logistic regression, age and BMI odds ratio (OR) for induction success were 0.795 and 0.857, respectively. The study population was divided into two groups based on age (<35 and ≥35 years) and obesity (BMI <30 and ≥30). Older women reported a higher induction failure rate (*p* < 0.001); longer time to cervical dilation of 6 cm (*p* = 0.03) and delivery (*p* < 0.001). Obese women reported a higher induction failure rate (*p* = 0.01); number of misoprostol doses (*p* = 0.03), longer time of induction (*p* = 0.03) to cervical dilatation of 6 cm (*p* < 0.001), and delivery (*p* < 0.001); and higher cesarean section (*p* = 0.012) and episiotomy rate (*p* = 0.007). In conclusion, maternal age and BMI are two of the main factors that influence oral misoprostol efficacy and affect the failure of induction rate in term PROM.

## 1 Introduction

Premature rupture of membrane (PROM) involves the rupture of membranes before the beginning of uterine contractions and labor. This condition occurs in 8–10% of pregnancies, and about 60% of them are at term (37 weeks or more of gestation) [1]. In the majority of term PROM, labor arises spontaneously within 24 h, even when the bishop is unfavorable [2]; however, approximately 40% of the cases will require more than 24 h [3]. In these cases, the labor may be delayed up to 7 days after PROM [2]. Term PROM is associated with immediate risks such as cord prolapse, cord compression, and placental abruption, as well as later complications such as maternal infections (chorioamnionitis and endometritis) and neonatal infections (neonatal sepsis) [4]. The risk of maternal chorioamnionitis and endometritis, which may result in subsequent neonatal infection, lung disease, and cerebral palsy as well as severe morbidity for the mother, increases proportionally with the time between the rupture of membranes and birth [5,6]. This risk factor was further confirmed by evidence that active management of term PROM by induction of labor results in a lower risk of maternal infectious morbidity (chorioamnionitis and/or endometritis) and neonatal sepsis compared with expectant management beyond 24 h, without an increased rate of cesarean section (CS) [3,7]. On that basis, induction of labor is the recommended strategy in these cases [8]. In term PROM, different methods are available to ripen the cervix and/or induce labor: the main important ones are intravenous oxytocin and oral or vaginal prostaglandins, which reported overall comparable effectiveness [7,9,10,11]. Prostaglandins are used for cervical ripening, but at the same time they commonly induce labor, making the distinction between ripening and labor induction almost artificial [9,10,11,12,13,14]. Prostaglandins are derivatives of arachidonic acid and are involved in different physiologic processes. Synthetic analogues of natural prostaglandins are available and utilized for their biological activities, including misoprostol (PGE1) and dinoprostone (PGE2). Although only dinoprostone (PGE2) was approved for cervical ripening/labor induction as cervical gel, intravaginal gel or tablet, misoprostol has been used off label for over 30 years as a labor-induction and cervical-ripening agent for the advantages of significantly lower cost, wide accessibility, and temperature stability compared to dinoprostone [9,13,14,15,16]. Furthermore, according to the latest Cochrane systematic review on the subject, low-dose oral misoprostol was associated with fewer cesarean deliveries, and consequent increase in vaginal deliveries, compared to vaginal dinoprostone, and lower rates of hyperstimulation with changes in the fetal heart rate [17]. Nevertheless, in 2014, generic misoprostol was licensed in Italy by the Italian agency of drug (Agenzia italiana del farmaco, AIFA) for induction of labor in term pregnancies and became available for every day clinical practice consistent with the protocol proposed by the World Health Organization (WHO) [18]. On that basis, the routine management for term PROM was to recommend cervical ripening and induction of labor with oral misoprostol when the bishop score is <7, instead of vaginal prostaglandins.

Although misoprostol has been used for over 30 years and the recent Cochrane review on oral misoprostol included 76 trials demonstrating the effectiveness of this induction method, the pieces of evidence are limited by the variety of different dose and time regimens proposed so far [14]. Moreover, little is known about factors in term PROM that may influence oral misoprostol efficacy and that may be related to induction failure. In this scenario, we conducted a retrospective cross-sectional study, based on prospectively collected data, including term pregnant women with PROM who underwent cervical ripening/induction of labor with oral misoprostol, consistent with the protocol proposed with the WHO [18]. Specifically, the aim of this study was to evaluate the effect of maternal age and body mass index (BMI) on oral misoprostol induction of labor for PROM at term.

## 2 Methods

This is a retrospective cross-sectional study of prospectively collected data. We used the oral misoprostol regimen proposed by the WHO [18] for induction of labor in term (37 weeks or more of gestation) singleton pregnancies in women who have not had a previous cesarean delivery and a bishop score <7. The regimen was oral misoprostol in an aqueous solution at a low dose of 25 µg every 2 h until a bishop score ≥7, labor, or for a maximum of 8 doses. Cardiotocographic (CTG) evaluation was conducted for at least 30 min before and continued for at least 60 min after each dose of oral misoprostol. Contraindications for the use or the continuation of oral misoprostol were labor (defined as the presence of at least three painful uterine contractions every 10 min), uterine tachysystole (>5 contractions within 10 min for two consecutive 10 min periods), hypertonic uterus, abnormal CTG, and contraindications to vaginal delivery (fetal malpresentation such as a breech presentation or transverse lie, fetal macrosomia, abnormally implanted placenta, active genital herpes infection, cervical cancer). Moreover, patients with parity >4, medical contraindications to misoprostol (asthma, glaucoma), and previous hysterotomies were excluded. In the case of term PROM, oral misoprostol was started at 24 h from PROM if the vaginal-rectal swab was negative for group B streptococcus (GBS), and after 6 h if positive. Moreover, prophylactic antibiotics were administered to prevent neonatal sepsis in cases in which PROM lasted >18 h if the vaginal tampon was negative for GBS and immediately if positive. In order to avoid potential biases, we included in the current analysis only women with vaginal-rectal swab negative for GBS.

Since the introduction of the oral misoprostol regimen in 2015, for all women who underwent labor induction with the new protocol, we prospectively recorded data about general characteristics (age, BMI, gestational age), patients’ history before pregnancy (previous surgeries, disease, therapies), course of pregnancy/recording maternal complications and fetal diseases, mode and characteristics of delivery, and fetal outcomes after birth (weight, Apgar, cord arterial blood pH, and base excess). Moreover, we recorded data about cervical ripening/labor induction such as bishop score (determined by assessing cervical dilation, effacement, station, position, and cervical consistency) at the first misoprostol dose, the number of doses administered, time of induction (time between the first dose and active labor, which was defined as regular uterine contractions and cervical dilation >4 cm), the time between last misoprostol dose and cervical dilation of 6 cm, time between last misoprostol dose and vaginal delivery, induction failure that was defined as an inability to generate regular contractions and cervical changes and/or without the onset of labor after eight doses of oral misoprostol, which required to continue induction with intravenous oxytocin or CS.

For the aim of this study, we retrospectively analyzed the prospectively collected database from January 2015 to June 2018. We included in the analysis pregnant women who underwent cervical ripening/labor induction with oral misoprostol with the following characteristics: nulliparous, single fetus, cephalic presentation, no use of any other cervical ripening methods within 7 days of hospitalization, PROM (diagnosed via vaginal speculum examination in order to determine the amniotic fluid leakage) between 37 and 42 weeks of gestation, and misoprostol started at 24 h from PROM. We considered the following as exclusion criteria: required oxytocin infusion to augment labor, hypertensive disorders, gestational diabetes, preconceptionally maternal diseases, concomitant fetal disease, or fetal birthweight <2,500 g or >4,500 g.

### 2.1 Outcomes and statistical analysis

Statistical analysis was performed using SPSS V.21.0 (IBM Corporation, Armonk, NY, USA). Descriptive statistics were reported according to data distribution as mean ± SD when they had a Gaussian distribution or median and interquartile range (IQR) when they were not normally distributed; the categorical variables were reported as absolute numbers and percentage (%). Binomial logistic regression was used to evaluate factors related to induction failure. Fisher’s exact test, and parametric (*t*-test, ANOVA) and non-parametric (Mann–Whitney, Wilcoxon) tests were used to compare baseline characteristics and outcomes as appropriate. Statistical significance was set for *p* < 0.05.

Based on a previous report (in line with our clinical practice) showing a mean of 5.5 h from the first dose of oral misoprostol to achieve active labor [19] and a potential 25% increase between women with BMI < 30 and BMI > 30, the inclusion of 41 women for each of these two arms would have been calculated to achieve a power of 80% with alpha error 0.05.

### 2.2 Ethics and methodological standards

All procedures performed in this study were in accordance with the ethical standards of the institutional and/or national research committee and with the 1964 Helsinki Declaration and its later amendments or comparable ethical standards.

The design, analysis, interpretation of data, drafting, and revisions conform to the Helsinki Declaration, the Committee on Publication Ethics (COPE) guidelines (http://publicationethics.org/), and the RECORD (reporting of studies conducted using observational routinely collected health data) statement [20], validated by the EQUATOR (enhancing the quality and transparency of health research) network (www.equator-network.org).

Each patient enrolled in this study signed an informed consent for all the procedures to allow data collection and analysis for research purposes. The study was non-advertised, and no remuneration was offered to encourage patients to give consent for the collection and analysis of their data. An independent data safety and monitoring committee evaluated the results.

The retrospective study design and development, with anonymized handling of the data, was approved by the Institutional Review Board of the study centers.

## 3 Results

Between January 2015 and June 2018, based on inclusion and exclusion criteria, 91 pregnant nulliparous women underwent induction of labor with oral misoprostol for PROM between 37 and 42 weeks of gestation. The mean study population age was 29.9 ± 5.6 years and the mean BMI was 30.1 ± 4.7. PROM occurred in the median at 40 (38–40) weeks of gestation (mean of 278.5 ± 8.1 days of gestation). Cervical ripening and induction of labor failed in 12 out of 91 (13.2%) pregnancies and required further induction with intravenous oxytocin to achieve labor or CS. Seventy-nine (86.8%) women underwent labor after oral misoprostol induction, 69 (87.3%) of them achieved vaginal delivery and 10 (12.7%) underwent CS for dystocia or non-reassuring CTG. The mean time of induction was 304.6 ± 133.6 min with a median of 5 (3–7) doses of oral misoprostol per patient. Cervical dilatation of 6 cm was achieved after a mean of 220.4 ± 241.2 min from the last misoprostol dose, and vaginal delivery was achieved after a mean of 357.2 ± 277.0 min. Episiotomy was performed in nine patients (9.9%), and 61 out of 69 (88.4%) vaginal delivery experienced perineal tears of the first or second degree. The mean fetal birthweight was 3216.3 ± 361.52 g. The median Apgar score was 9 for both the first (8–9) and fifth (9–10) minutes. The mean fetal cord blood pH and base excess were 7.24 ± 0.08 and −5.6 ± 3.02, respectively. No newborn was admitted to the neonatal intensive care unit.

We performed a multivariate logistic regression to evaluate factors that may influence oral misoprostol efficacy and that may be related to the failure of induction in term PROM. We investigated the effect of gestational age (days), maternal age (years), BMI (kg/m^2^), and fetal weight on induction success, and the results of logistic regression are reported in Table 1. Age and BMI results were associated with the risk of oral misoprostol induction failure. The age reported an odds ratio (OR) of 0.795 (95% confidence interval [CI]: 0.679–0.931) per each year of increased age for induction success; similarly, BMI reported an OR of 0.857 (95% CI: 0.737–0.997) per each unit of BMI for induction success.

**Table 1:** Binomial logistic regression for factors influencing the success of labor induction with oral misoprostol in women with term PROM

	*P*	OR	90% CI – inferior OR	90% CI – superior OR
Gestational age (days)	0.873	0.993	0.905	1.088
Maternal age (years)	**0.004**	0.795	0.679	0.931
BMI (kg/m^2^)	**0.045**	0.857	0.737	0.997
Fetal birthweight	0.672	1.000	0.998	1.001
Constant	0.240	43569382.670		

Values shown in bold are those that reached statistical significance.

Based on these results, the study population was investigated by dividing patients based on age and BMI. Table 2 reports data on the study population analyzed after dividing it into two groups based on the cutoff of 35 years old. This analysis confirms the higher induction failure rate in women older than 35 years (OR = 13.9; *p* < 0.001); moreover, the results reported a longer time to achieve a cervical dilation of 6 cm (*p* = 0.03) and delivery (*p* < 0.001) after the last oral misoprostol dose. Based on BMI (Table 3), the study population was divided into two groups based on the diagnosis of obesity (BMI ≥ 30) or non-obesity (BMI < 30): obese women reported a higher induction failure rate (OR = 7.3; *p* = 0.01), number of oral misoprostol doses (*p* = 0.03), time of induction (*p* = 0.03), cervical dilatation of 6 cm (*p* < 0.001) and delivery (*p* < 0.001); moreover they reported a higher CS rate (*p* = 0.012) and episiotomy rate (*p* = 0.007) compared with non-obese women.

**Table 2:** Comparison between women aged <35 or ≥35 years with PROM who underwent labor induction with oral misoprostol

	PROM <35 years (*n* = 68)	PROM ≥35 years (*n* = 23)	*p*
Maternal age (years)	27.48 ± 4.05	37.13 ± 2.33	**<0.001**
Gestational age (days)	278.53 ± 8.17	278.61 ± 7.61	0.97
BMI (kg/m^2^)	29.57 ± 4.44	31.78 ± 5.62	0.06
Misoprostol doses	5 (3–7)	7 (4–7)	0.092
Time of induction (min)	291.18 ± 133.73	344.35 ± 130.12	0.10
Time between the last misoprostol dose and cervical dilation of 6 cm (min)	189.74 ± 191.04	381.82 ± 398.32	**0.003**
Time between the last misoprostol dose and delivery (min)	316.12 ± 229.41	573.64 ± 398.51	**<0.001**
Induction failure	3/68 (4.4%)	9/23 (39.1%)	**<0.001**
CS	7/65 (10.8%)	3/14 (21.4%)	0.37
Episiotomy	7/58 (12.1%)	2/11 (18.2%)	0.63
II-degree vaginal laceration	24/58 (41.4%)	7/11 (63.6%)	0.20
Neonatal weight (g)	3,196.98 ± 367.12	3,273.47 ± 360.09	0.39
Cord arterial blood pH	7.23 ± 0.09	7.26 ± 0.07	0.15
Cord arterial blood base excess	−5.95 ± 3.20	−4.57 ± 2.19	0.16
Apgar at 1 min	9 (8–9)	9 (8–9)	0.67
Apgar at 5 min	9 (9–10)	9 (9–10)	0.27

Values shown in bold are those that reached statistical significance.

**Table 3:** Comparison between non-obese (BMI <30) and obese (BMI ≥30) women with PROM who underwent labor induction with oral misoprostol

	PROM <30 BMI (*n* = 49)	PROM ≥30 BMI (*n* = 42)	*p*
Maternal age (years)	29.10 ± 5.44	30.88 ± 5.83	0.13
Gestational age (days)	280.35 ± 7.46	278.45 ± 8.30	0.25
BMI (kg/m^2^)	26.37 ± 1.60	34.52 ± 2.99	**<0.001**
Misoprostol doses	5 (3–6.5)	6 (4.75–7)	**0.03**
Time of induction (min)	276.73 ± 132.05	337.14 ± 131.97	**0.03**
Time between the last misoprostol dose and cervical dilation of 6 cm (min)	117.89 ± 105.24	412.5 ± 307.79	**<0.001**
Time between the last misoprostol dose and delivery (min)	251.89 ± 164.43	554.58 ± 344.40	**<0.001**
Induction failure	2/49 (4.1%)	10/42 (23.8%)	**0.01**
CS	2/47 (4.3%)	8/32 (25%)	**0.012**
Episiotomy	2/45 (4.4%)	7/24 (29.2%)	**0.007**
II-degree vaginal laceration	17/45 (37.8%)	14/24 (58.3%)	0.13
Neonatal weight	3,227.86 ± 361.47	3,202.86 ± 368.02	0.74
Cord arterial blood pH	7.24 ± 0.09	7.24 ± 0.08	1
Cord arterial blood base excess	−5.68 ± 2.31	−5.51 ± 3.56	0.78
Apgar at 1 min	9 (8–9)	9 (8–9)	0.48
Apgar at 5 min	9 (9–10)	9 (9–10)	0.46

## 4 Discussion

Our results show that maternal age and BMI are two of the main factors that influence oral misoprostol efficacy and that are related to the failure of labor induction in term PROM. Both logistic regression and subsequent analysis confirm a higher risk of induction failure and a longer time to achieve cervical dilatation and delivery after induction for term PROM with oral misoprostol in older women. At the same time, obese women required a higher number of oral misoprostol doses, with subsequent longer time of induction, reporting a higher failure rate; moreover, obese women had longer labor and a higher rate of CS and episiotomy.

A higher risk of induction failure and CS after induction were previously reported in older women [21,22]. Similarly, obesity was already associated with a higher risk of induction failure, a higher risk of CS, and prolonged labor [23,24] due to impaired uterine contractility, potentially caused by altered cholesterol levels, increased leptin concentration, and hormonal imbalance [25].

We acknowledged that available pieces of evidence about the induction of labor using oral misoprostol report different and sometimes conflicting results. This variability can be explained, at least in part, by the different doses (from 20 to 200 µg) used for the induction of labor in the different studies published so far [26].

To the best of our knowledge, this is the first study demonstrating that both age and BMI play a key role in influencing oral misoprostol efficacy and failure rate of labor induction in term PROM.

This evidence may solicit to investigate the effectiveness of different available methods in older and obese women, considering that different pharmacokinetics and pharmacodynamics could support the use of a method instead of another one in these subpopulations. For example, misoprostol is available to be administered in oral, sublingual, and vaginal routes; nevertheless, pharmacokinetic studies suggest that vaginal misoprostol leads to a lower peak serum concentration but delayed time to peak concentration compared with oral administration, with greater exposure to the drug when it is vaginally administered [27]. Moreover, considering the heterogeneity of different dose and time regimens of oral misoprostol available in the literature [14,28], our study may suggest investigating a higher dosage and/or different time of oral misoprostol in obese and older patients instead of other induction methods. Finally, in everyday clinical practice, our results may help clinicians in counseling patients about the failure rate and time of induction and delivery.

Although this study gives new insight into the cervical ripening/labor induction in term PROM with oral misoprostol, our results are limited by the retrospective study design, which is intrinsically the carrier of bias compared to the prospective approach and by the limited number of patients included in the study population. Nevertheless, the prospectively collected database, the standard applied protocol, and the strict inclusion and exclusion criteria provide robust results and limit this bias. Despite the relatively low number of women enrolled, this is fully within the sample size analysis, so the study can be considered sufficiently powered to detect significant differences between the groups.

In particular, we included only term PROM in healthy nulliparous women with single cephalic fetuses with normal birthweight and uneventful pregnancy that were induced 24 h after PROM; moreover, the exclusion of women further induced with oxytocin or with oxytocin-augmented labor was mandatory to avoid the bias of oxytocin action.

In our opinion, however, it would be appropriate to perform further clinical analyses aimed to evaluate women further induced with oxytocin or oxytocin-augmented labor after primary induction with oral misoprostol in an aqueous solution, in order to address future research priorities.

In conclusion, our study shows that maternal age and BMI are two of the main factors that influence oral misoprostol efficacy for labor induction in term PROM. Older and obese women have a higher risk of induction failure and have poorer responses to oral misoprostol. These data may help to perform detailed counseling and may guide further research on different dose and time regimens of oral misoprostol or different induction methods in these subpopulations. Based on these preliminary data, we take the opportunity to solicit further studies to investigate the possible factors influencing the effectiveness of cervical ripening/induction of labor in term PROM.
